# In vitro Interactions between *Streptococcus intermedius* and *Streptococcus salivarius* K12 on a Titanium Cylindrical Surface

**DOI:** 10.3390/pathogens9121069

**Published:** 2020-12-20

**Authors:** Carla Vacca, Maria Paola Contu, Cecilia Rossi, Maria Laura Ferrando, Cornelio Blus, Serge Szmukler-Moncler, Alessandra Scano, Germano Orrù

**Affiliations:** 1 Molecular Biology Service (MBS), Department of Surgical Sciences, University of Cagliari, 09124 Cagliari, Italy; cvacca86@gmail.com (C.V.); contu_paola@tiscali.it (M.P.C.); cecilia.rossi.uni@hotmail.it (C.R.); laura.ferrando@wur.nl (M.L.F.); corblus@hotmail.com (C.B.); ssm@bluewin.ch (S.S.-M.); alessandrascano@libero.it (A.S.); 2 Private Dental Practice, 22100 Como, Italy; 3 Excellence in Dentistry (EID) Research Group, 36, Rue de Lubeck, 75116 Paris, France; 4 National Research Council of Italy, 07100 Sassari, Italy; 5 Azienda Ospedaliero Universitaria (AOU) Cagliari, 09124 Cagliari, Italy

**Keywords:** peri-implantitis, *Streptococcus salivarius*, *Streptococcus intermedius*, biofilm, *luxS*

## Abstract

Peri-implantitis is a steadily rising disease and is caused by oral bacterial pathogens able to form biofilm on implant surfaces and peri-implant tissues, making antibiotics treatment less effective. The use of commercial probiotics against oral pathogens could serve as an alternative to prevent biofilm formation. *Streptococcus intermedius* is one of the early colonizers of biofilm formation in dental implants. The aim of this study was to model the interaction between *S. intermedius* and *Streptococcus salivarius* strain K12, a probiotic bacterium producing bacteriocins. *S. intermedius* was co-cultured with *S. salivarius* K12 in an in vitro model simulating the biofilm formation in a dental implant composed by a titanium cylinder system. Biofilm formation rate was assessed by Real-Time PCR quantification of bacterial count and expression levels of *luxS* gene, used in response to cell density in the biofilm. Biofilm formation, bacteriocin production, *luxS* expression patterns were found to be already expressed within the first 12 h. More importantly, *S. salivarius* K12 was able to counter the biofilm formation in a titanium cylinder under the tested condition. In conclusion, our dental implant model may be useful for exploring probiotic-pathogen interaction to find an alternative to antibiotics for peri-implantitis treatment.

## 1. Introduction

The implementation of dental implants is due to edentulism (partial or complete absence of teeth), which causes the loss of masticatory function. Different materials have been used to construct dental implants over many centuries. In the last decades, titanium is considered the most commonly used material to replace missing teeth. The main alloys are made in commercially pure titanium (cpTi) and Ti-6Al-4V, and it represents one of the most durable materials in implantology [1,2] Titanium-based alloys have shown numerous benefits including high biocompatibility and durability, with a success rate of up to 99% in oral implantology registered in the last 10 years. Despite these advantages, the dental implants can become infected from oral pathogen bacteria, causing a chronic inflammatory process in soft tissues and around the osseointegration implant [3]. For this reason, infections remain a major reason for dental implant failure. 

Peri-implantitis (PI) is a disease that is on the rise in dental implantology. The PI disease is primarily classified as mucositis when peri-implant soft-tissues are affected and then labelled peri-implantitis when progressive bone loss sets in. Recent reports indicate that the percentages of clinically evident PI range from 28 to 56%, while peri-implant mucositis reaches about 80% [4,5]. PI infection is caused by oral bacteria pathogens colonizing the peri-implant tissues and begins with bacterial adhesion to the implant surface followed by a proliferation of multispecies bacteria resulting in a polymicrobial community.

In a recent article, Maruyama et al. [6] observed a substantial difference in the microbial profile of bacteria collected from the dental plaques of patients with both peri-implantitis and periodontitis. The bacterial biofilm motif is thought to be responsible for the failure of PI antimicrobials treatments by preventing the penetration of antibiotics in the infection site [7]. Streptococcal genus plays a crucial role in biofilm formation [8,9] being pioneer colonizers can also drive the bacterial community composition of the oral biofilm [10]. In addition, the oral bacterial community can influence the health/disease status of the host by communicating with human cells using small Quorum Sensing signals (QS) molecules [11].

Some streptococcal species/strains are characterized by a high virulence while others show a mutualistic relationship with the host; whilst other non-pathogenic *Streptococcus* display potential probiotic properties [12]. Two streptococcal species, such as *Streptococcus intermedius* and *Streptococcus salivarius*, provide a representative example of pathogen/commensal discrepancy in the oral cavity.

*S. intermedius* is a systemic pathogen implicated in severe human infections including endocarditis, pneumonia, and tissue abscesses [13,14,15,16]. It plays a primary role as biofilm initiator being an early colonizer of dental implants surfaces, especially in biofilm-associated diseases such as periodontitis and PI [17,18]. It has been demonstrated that QS mediated by synthetic Competence-Stimulating Peptides (CSP) was associated with the development of biofilm mode without affecting the rate of *S. intermedius* growth [19]. 

*The luxS* gene is involved in *luxS*/AI-2 QS system and synthetizes AI-2 (autoinducer-2) as signalling molecule. AI-2 mediates both intra- and inter-species communication and triggers genes regulatory cascades which modulate various cellular processes., *luxS* has been identified in both Gram-negative and Gram-positive bacteria and it takes part in one of the global regulatory networks in bacteria. It responds to fluctuations of bacterial density and regulates the expression of a number of genes, influencing numerous cell behaviours such as biofilm formation. In this context different levels of *luxS* expression patterns are linked to the ability of a bacterial community to biofilm-forming, as well as the drug resistance profile [20].

In *S. intermedius luxS*/AI-2 has also been found to influence biofilm formation and virulence in response to cell population density [21] and various external stimuli, such as antibiotic treatment [22]. 

*S. salivarius* is part of the healthy core oral microbiota in humans and shows great potential as a probiotic targeting the oral flora dysfunction. *S. salivarius* strain K12 was isolated from the throat of a healthy child from New Zealand and produced two forms of lantibiotics (salivaricin A2 and B). The specific strain is commercially available and used for bacterial therapy in paediatric pharyngitis [23]. More recently, its administration has been considered as a possible alternative cure in oral halitosis and periodontal diseases [24,25]. Previous research demonstrated no adverse reaction in subjects orally treated with strain K12 [26,27]. Microbiological and virulence gene profiles have indicated that this bacterium has a low virulence potential [28]. The potential use of *S. salivarius* could be also considered in bacterial therapy to contrast the development of biofilm formed by *S. intermedius*. However, very little is known about the physical interaction between these two oral bacteria during biofilm formation. More specifically, we lack information on their growth ratio, the amount rate and time of mature pathogen biofilm formation, and the time of inhibition in the presence or absence of the probiotic counterpart. We hypothesized: (i) bacterial colonization could occur immediately after implant placement; (ii) the probiotic bacterium *S. salivarius* could compete, in the biofilm formation, with the pathogen *S. intermedius*, reducing the risk of peri-implantitis.

The aim of our study is, therefore, to present an in vitro model able to evaluate the kinetics of interaction of the *S. intermedius/S. salivarius* binary system mimicking a PI in vitro model.

## 2. Results

### 2.1. Protein Coating Rate on the Titanium Cylinder Surfaces

**Figure 1:**
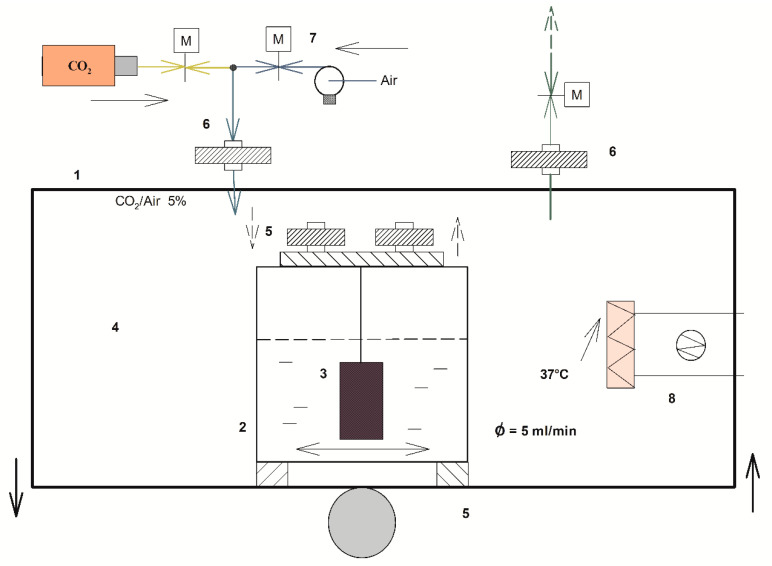
DIB, Dental Implant Bioreactor. (1) In vitro model of the customized culture system used to study the interaction between the oral pathogen *S. intermedius* and the commensal *S. salivarius* K12. (2) Glass flask containing the SB culture medium achieved mixing Saliva/Shaedler broth with a proportion of 8:2. (3) Titanium cylinders placed in the tube. (4) Thermostatic chamber kept at 37 °C. (5) Shaking support. (6) Air filters. (7) CO_2_/air mix. (8) Electrical resistance. The medium flow (ф) was kept at 5 mL/min to simulate the normal saliva flux. The bioreactor was positioned on a shaker, producing vertical oscillations at a frequency of around 10 rpm/min.

**Figure 2:**
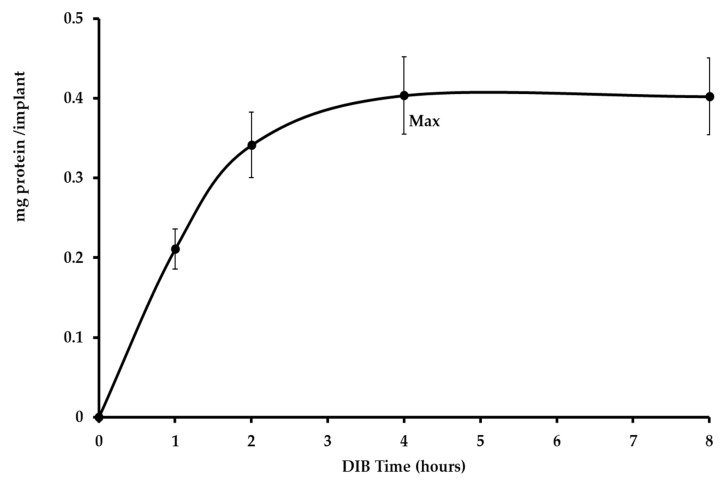
Kinetics of protein surface formation in eight hours in the titanium cylinder. The maximum amount of protein slime was observed at 4 h (an average of 0.40 mg/implant), *S. intermedius* and *S. salivarius* were inoculated into the DIB bioreactor during this time.

Protein pellicles derived from saliva play a significant role in the progression and maintenance of microbial colonization of surfaces, especially during the first phase of biofilm formation mediated by early bacteria colonizers, such as oral Streptococci [29,30]. The total protein amount mg/total was collected from the cylinder surface of Dental-implant Bioreactor (DIB) (Figure 1, see Material and method) and measured at different incubation times. Figure 2 represents the kinetics of the protein deposition on the implant surface during the experiment. The maximum protein deposition was observed after 4 h of incubation in the DIB with SB medium, after which the protein content stayed constant for other 4 h.

For this reason, *S. intermedius* was inoculated into the bioreactor after 4 h of incubation when the average amount of protein slime reached the maximum concentration with 0.4 mg/total protein/cm^2^. In fact, the interactions between salivary components in the pellicles and micro-organisms affect initial microbial adherence [31] and this process thus resulted as essential i.e., for plaque formation and other biofilm-associated diseases [32,33].

### 2.2. Kinetics of S. intermedius Biofilm in the Titanium Cylinder

**Figure 3:**
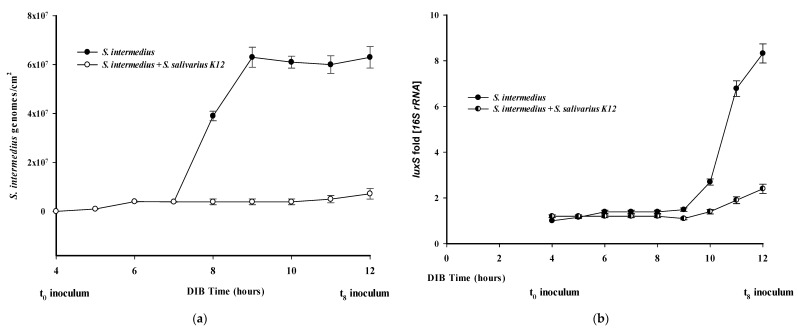
S. intermedius adhesion rate and related luxS gene expression pattern. (**a**) Curve relative to *S. intermedius* adhesion to the titanium cylinder. (**b**) *luxS* expression rate. The max. expression values were observed at 8 h from bacterial inoculum, 12 h from experiment start.

After the inoculation of *S. intermedius* into the DIB, we measured at different times the *S. intermedius* genomes copies deposited on the titanium cylinder coated with salivary proteins. The bacterial adhesion was detectable about in the first 4 h after inoculum with a maximum biofilm concentration after 5 h (6.1 × 10^7^ bacterial genomes/cm^2^), (Figure 3a). Moreover, from 0 to 8 h after *S. intermedius* inoculum, we evaluated an expression rate of the *luxS* gene (max 8.3 folds at t_8_ vs t_0_), (Figure 3b). These results indicated that after 8 h the pathogen *S. intermedius* was already structured in an actively growing biofilm around the cylinder surface. As shown in Figure 3a, the presence of the probiotic strain resulted in a decrease in *S. intermedius* of approximately 1 log_10_ less) folds after 12 h of incubation (from 7.1 × 10^7^ to 6.0 × 10^6^ genomes/cm^2^, respectively). In these experimental conditions, the period 8 to 12 h, from experiment start, represented the number of major events for implant colonization by *S. intermedius*. In addition, the presence of the probiotic strain *S. salivarius* caused a low *luxS* expression pattern in *S. intermedius*, (Figure 3b).

### 2.3. S. salivarius Growth Curve and Bacteriocin Activity in the Bioreactor

**Figure 4:**
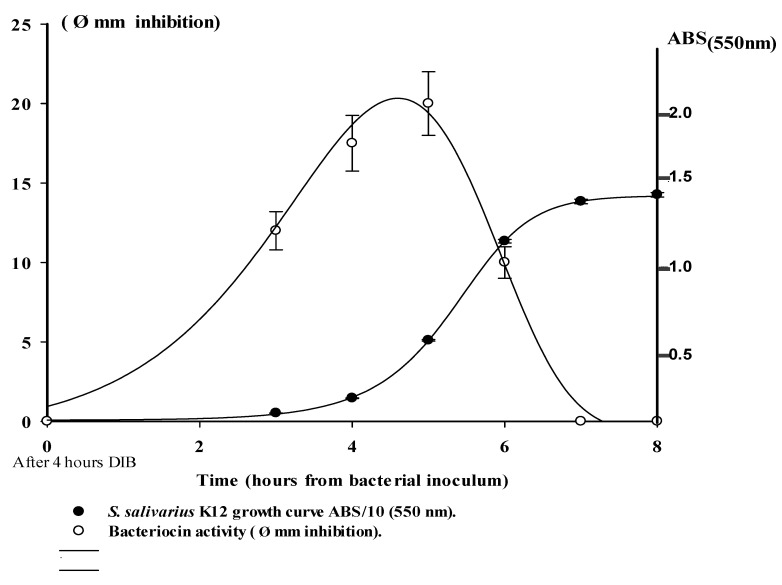
Comparison of bacteriocin activity and growth curve in *S. salivarius* K12 strain inoculated into SB medium in a DIB.

The bacteriocin activity of *S. salivarius* was measured in DIB medium in an in vitro model to verify whether the expression of bacteriocins produced by *S. salivarius* could exert any inhibitory activity against the mature biofilm formation mediated by *S. intermedius*. The antibacterial activity was detected from 2 to 6 h of growth of *S. salivarius* and persisted until the *S. salivarius* had reached the med log-growth phase in the DIB at about 6 h (Figure 4). The adhesion of *S. salivarius* to the components of the DIB followed the same kinetics as *S. intermedius*, with a maximum amount of bacterial deposition on the surface of the titanium cylinder after 6–8 h from the *S. salivarius* inoculum (data not shown).

The antimicrobial activity of *S. salivarius* was confirmed testing the culture medium filtrate by microplate growth inhibition assay using serial dilutions of the medium (see method). Table 1 shows the respective values for Minimum Inhibitory Concentration (MIC), CMB, and Minimum biofilm Inhibitory Concentration (MBIC) for the *S. intermedius*. In these conditions, the antimicrobial activity was ranged from 50% (MIC) and 12.5% (MBIC) as medium dilution, while the minimum bactericidal concentration (MBC) was higher than 50%. Sterile medium did not show any antimicrobial activity (control).

### 2.4. Effect of the Probiotics on the S. intermedius Biofilm

To test whether *S. salivarius* K12 could antagonize the adhesion of *S. intermedius* it was inoculated with *S. intermedius* (1 × 10^6^ CFU/mL each) to the DIB medium after 4 h from experiment start and the amount of copies genomes of both bacteria were measured in a period between 4 and 12 h.

A reduction of the 87% of *S. intermedius* number (genomes/cm^2^) was observed when the probiotic *S. salivarius* was added to the bioreactor medium titanium cylinder, (Figure 3a). In fact, without probiotic treatment, at 8 h of incubation time, 12 h from experiment start, the mean of the final titre was 6.3 × 10^7^ genomes/cm^2^, whereas, in presence of *S. salivarius*, the final amount was 7.9 × 10^6^ genomes/cm^2^. This result suggests an active effect of the probiotic strain on *S. intermedius* biofilm structuration, able to reduce the number of cells already attached to the surface (*p* < 0.05).

**Figure 5:**
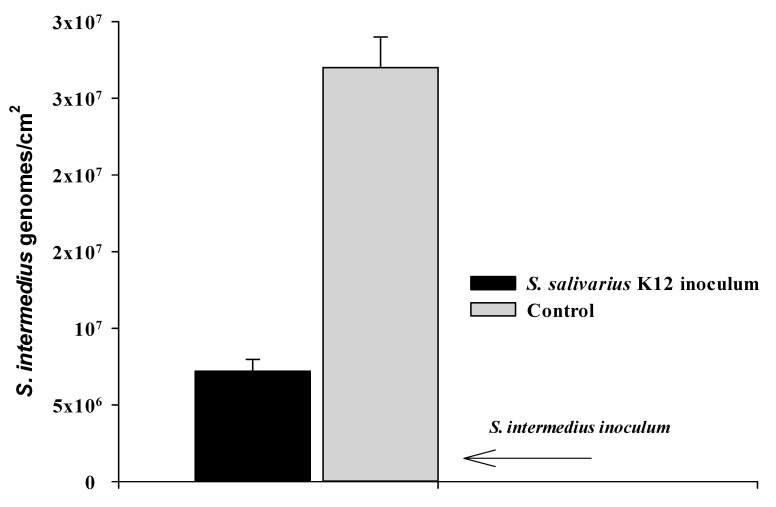
Influence of *S. salivarius* K12 on the *S. intermedius* after 20 h incubation. The biofilm is expressed as n. genomes/cm^2^ titanium cylinder surface. The graph express biofilm means values and the standard errors for three samples in three independent experiments.

**Figure 6:**
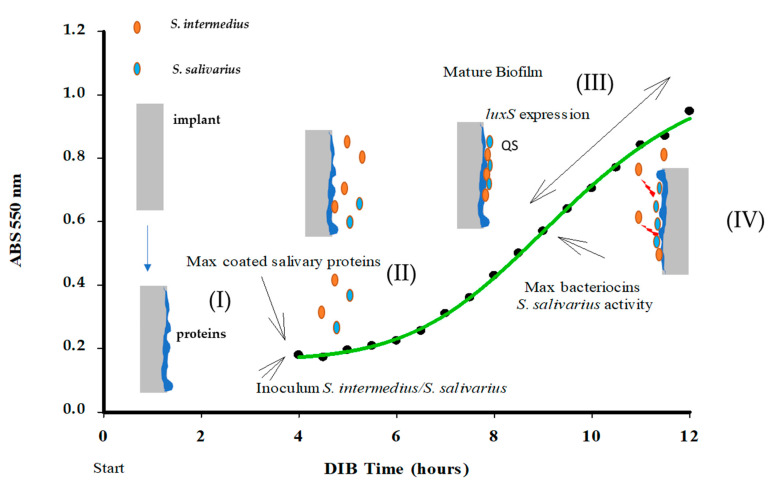
The experimental scenario representing *S. intermedius* behaviour on the titanium surface and the interaction with the probiotic bacterium *S. salivarius K12*. (**I**) Protein surface coating, (**II**) Inoculum of two bacteria species in the DIB system, (**III**) Bacterial Biofilm formation on the titanium surface of the DIB after 8 h of incubation (**IV**) Max production of bacteriocins by *S. salivarius* acting against *S. intermedius,* Max expression of *luxS* mRNA 8 h from, 12 h from experiment start.

This effect is also measurable after a long time of incubation with two Streptococcal strains, in fact as reported in Figure 5 a substantial difference is observed a 24 h of DIB growth, after 20 h of *S. intermedius-S. salivarius* inoculum, 2.7 × 10^7^ genomes/cm^2^ vs. 7.1 × 10^6^ genomes/cm^2^, i.e., 74% in biofilm reduction (Figure 5). In addition, this interference on *S. intermedius* biofilm development by probiotic is also observable by a considerable inhibition of pathogen *luxS* gene expression, as reported in Figure 3b. Figure 6 illustrates the crucial times evaluated in DIB and compared with the growth curve of *S. salivarius*.

## 3. Discussion

Peri-implantitis and peri-mucositis are considered a major and increasing problem in dentistry and, despite the current lack of information on its exact prevalence, recent reports have suggested a frequency of around 50% in the western population [34,35]. Although new therapeutic procedures may be in the pipeline against this disease, biofilm-related infections cannot be entirely cured [35,36,37,38]. In addition, possible implant failure or a large number of available treatments to avoid clinical complications are often costly [39,40,41,42]. Implant-related infection represents a complex biological process starting with microbial adhesion and subsequent biofilm formation. The core of this infection is initially represented by early colonizer bacteria, which include mainly Streptococci [29,30,31]. In this context, different streptococcal species play a critical role in the succession of biofilm and in their overall effect on the oral-systemic health of the patient [43,44]. Being the bacterial adhesion to the host cells a multifactorial process, it has been found that many streptococcal cell-wall anchor proteins contribute to the binding of host-cells components [45,46,47,48]. Bacterial adhesins in the oral cavity enable interactions with salivary components [49], such as glycoproteins, host cell and other bacteria, which play a crucial role in this first step of colonization and, in this context the bacteria communication network represents another biological phenomenon required in biofilm formation through QS Signals. Other authors have studied the dynamic deposition of oral biofilm promoted by the cooperation of the oral commensals through QS system; here for the first time, we investigated the interaction of two antagonist oral bacteria to prevent oral biofilms formation, which represents one of the main risk factors in the development of bacterial infections of the oral cavity [50]. Actually, mature biofilm is characterized by a complex community of bacteria including some potential pathogens which may detach from the biofilm and spread into host tissues [51]. A flow chamber model for the assessment of the biofilm formation by multiple oral bacteria on implant materials has also been proposed by other researchers [8,52,53]. In this work, we reconstructed an implant infection archetype by using a titanium cylinder to study the mature biofilm formation of the oral pathogenic *S. intermedius*, which one the main agent responsible for PI and periodontal disease. Moreover, we intended to study, as possible preventive cure in PI, the interaction of probiotic-pathogen bacteria in our biofilm formation model. For this, we added the probiotic strain *S. salivarius* to the culture to evaluate whether this bacterium was capable of reduce the pathogen from the mature biofilm. Bacteriocin production from the probiotic strain and probiotic-pathogen interactions, such as biofilm amount and QS (*luxS*) signal were assessed in our PI mimicking model. Strikingly, *S. intermedius* could produce a bacterial biofilm around titanium cylinder in a short time (9–12 h from experiment start) and high amount (~10^7^ genomes/cm^2^). Previous works have demonstrated that the respective *luxS* in different bacteria, including *S. intermedius*, is an AI-2 synthase enzyme involved in the modulation of biofilm formation [54]. We speculated that the *luxS* expression rate could be an interesting parameter for the evaluation of the of the bacterial biofilm inhibition deposited on a dental cylinder surface, implying that AI-2 production represents the a *conditio sine qua non* for sessile life in *S. intermedius*. In a previous study, it has been shown that a complete monomicrobial biofilm was well detectable in about 10 h, of which 4 h for a complete protein coating and 6 h for the initial *S. intermedius luxS* gene expression essential in biofilm formation by a cell-cell communication system [55,56]. In our model, the expression of *luxS* gene reached the highest levels after 6–8 h from the initial inoculum (about 8-fold at t_8_ with respect to the t_0_ signal, (Figure 3b). These results are in accordance with the study described by Pecharki et al. in which the production of the *luxS* dependent autoinducer AI-2, measured by the level of bioluminescence induced in *Vibrio harveyi* reached a max. level at 5 h, even under different bacterial growth conditions [57].

In this implant in vitro infection model, we have investigated mainly two aspects: (a) the kinetic of microbial community development on a titanium surface, and (b) the potential role of an oral probiotic strain in the inhibition of the growth of pathogens biofilm-producer. 

We propose a model reporting of interaction between the bacteria in DIB displaying the kinetic parameters bacterial growth curve, pathogen QS signals, and probiotic bacteriocin production, (Figure 6). The inhibitory effect of the probiotic strain against *S. intermedius* biofilm is evaluable between the 6th−7th hours after inoculum when the presence of *S. salivarius* was able to interfere with the pathogen’s growth on the titanium cylinder, in the absence of its adhesion phase. This is in accordance with the bacteriocin production rate, observed from the 2nd and 6th hour in the *S. salivarius* growth curve (Figure 3a,b, Figure 4 and Figure 5).

These results show that bacteriocin production by probiotic bacteria appears crucial for the inhibition of dental implant biofilm, which is in accordance with other authors who have described the regulatory effect of bacteriocins in polymicrobial biofilms [58,59]. The probiotic strain *S. salivarius* is currently used in clinical practice in the prevention of different diseases of the human oro-respiratory tract and it is considered a pioneer commensal member of healthy oral microbiota [60]. The strain K12, also known as BLIS K12™, was initially selected to antagonize *Streptococcus pyogenes*, the most important bacterial cause of pharyngeal infections [61]. *S. salivarius* K12 is intended in the application as probiotic use due to the production of two lantibiotics salivaricins with a broad spectrum of antibacterial activity and its colonization capacity in the upper respiratory tract characterized by a long persistence on the tonsils. In addition to this, it proved to be a harmless bacterium with immunomodulatory activity of the host defence. For these features, it is currently used as a therapy against several pathogens associated with pharyngitis, otitis media, tonsillitis, halitosis, and it seems to reduce tracheitis, viral pharyngitis, rhinitis, flu, laryngitis, acute otitis media, and enteritis in children. There is also some in vitro and in vivo evidence of its ability to counter oral candidiasis.

Given our findings on the effectiveness of *S. salivarius* K12 in combatting an early implant biofilm colonizer as *S. intermedius*, the use of this probiotic could be considered in non-surgical therapy to prevent biofilm-related implant diseases.

## 4. Materials and Methods

### 4.1. Strains and Cultural Conditions

*S. intermedius* strain DSMZ 20573 obtained from the Leibniz Institute DSMZ. *S. salivarius* strain K12 (ATCC strain BAA-1 024) was isolated from a commercial product (Bactoblis®, PharmExtracta S.p.A, Pontenure Italy and BLIS Probiotics, Dunedin New Zealand). Bacteria were stored at −80 °C in Schaedler broth (Microbiol, Italy) plus 20% glycerol. Both strains were grown in the same broth at 37 °C in presence of 5% CO_2_ to mid-log-phase (OD_550_ = 0.125 or 0.5 McFarland) equal to 1.5 × 10^8^ CFU/mL, which was used as 100× inoculum.

To measure the biofilm formation, we used a modified method already described by Denotti et al, 2009. Briefly, saliva/broth medium was performed by mixing human saliva and Schaedler broth with ratio 8/2 (v/v) (SB medium) [62]. Unstimulated whole saliva was collected by the spitting method from six healthy subjects aged from 20 to 40 years and recruited from the Department of Dental Disease Prevention (University of Cagliari) and stored at −80 °C. All volunteers signed an informed consent form before taking part in the microbiological analysis. To avoid any proteins denaturation or salt precipitation in the saliva sample, bacteria were grown in 8 media/saliva mixture within max 24 h. Media/saliva mixture containing >20% of saliva caused a substantial delay in the growth for both strains (i.e., lag phase was reached after 12–15 h of incubation).

Saliva samples were thawed, clarified by centrifugation at 4000× *g* and, after pooling the samples together, were further centrifuged with the same speed for 10 min. The supernatant was then filtered with 0.45 μm diameter filter (Merk Millipore, Darmstadt, Germany) to eliminate any bacterial and cell residual. The filtrate was diluted in SB to obtain 80% saliva media and sterilized by filtration (filter 0.45 Ø μm, (Merk Millipore, Darmstadt, Germany). 200 mL of SB were aliquoted in each sterile tube contained in the bioreactor (Figure 1).

### 4.2. Peri-implantitis Bioreactor Model 

Bioreactor model was built as described by Denotti et al., 2009 with some changes for simulating a model of periiplantitis.

Dental-implant Bioreactor (DIB) model was reconstructed in a sterile flask containing a sterile titanium cylinder (99% titanium cylinder 15 × 5 mm Ø (Biaggini Medical Devices, Italy) to simulate a base dental implant as reported in Figure 1. The use of a smooth titanium cylinder was performed to avoid differences in microbial adhesion due to different implant designs and surface roughness showed from different manufactures [63]. Bacterial culture was protracted inside the DIB for 24 h at 37 °C in presence of 5% CO_2_ under shaking [64]. Every 30 min, 5 mL of bacterial culture were collected from the DIB model for, bacterial growth curves and bacteriocin activity assay. After each sampling, 5 mL of sterile SB medium was added into the bioreactor chamber.

The bioreactor was positioned on shaker generating vertical oscillations (Continental Instruments, Milan, Italy), (10 vertical oscillations/min) to avoid the adhesion of non-specific bacteria and reproduce the salivary flow of ≃5 mL/min (Figure 1). Each microorganism was inoculated into the bioreactor after the formation of coated implant protein pellicles, as described later. 

### 4.3. Bacteriocin Activity Assay

Bacteriocin activity from *S. salivarius* K12 was obtained following the agar plate diffusion method described by other authors [65]. 0.02 mL of *S. salivarius* filtered culture with Millipore 0.22 µm was put on a sterile paper disc (Whatman, Ø 6 mm, Merck Sigma-Aldrich, Darmstadt, Germany) and positioned in blood agar plate (Microbiol Uta Cagliari, Italy), previously inoculated with *S. intermedius* 1 × 10^7^ CFU/mL. After 24 h at 37 °C and 5% CO_2_, the diameter of the inhibition alone was measured. The negative control was obtained through the same procedure by the deposition of a sterile SB broth culture. Each assay was triplicated over three independent experiments.

#### Bacteriocin Microplate Assay (Microplate Growth Inhibition Assay)

To confirm the data on the antimicrobial-antibiofilm effect of *S. salivarius* against *S. intermedius* a microplate evaluation was performed. We have performed an experiment by using a liquid 0.45 um filtrate of SB medium obtained after *S. salivarius* growth, for 4 h at 37 °C with [5%] CO_2_. This medium was serially 1/2 diluted (from 50% to 1%) in a 96 well microplate containing SB medium and an inoculum of *S. intermedius* of 1 × 10^6^ CFU/mL. Minimum Inhibitory Concentration (MIC), Minimum Bactericidal Concentration (MBC), and Minimum biofilm Inhibitory Concentration (MBIC) were performed in accordance with already published works. In particular, for the biofilm evaluation, we used the protocol described by Montana University’s Canter for Biofilm Engineering, following the Chrystal Violet staining protocol [66,67].

### 4.4. Growth Curves

A growth curve was performed for both strains inoculating 1 × 10^6^ CFU/mL into the DIB containing sterile SB medium [67]. The growth rate of bacterial strains inside the bioreactor was measured at OD 550 nm with an incubation time of 12 h. In this time range, both bacteria strains showed a log growth phase, in these conditions the relationship between CFU/mL counting and optical density was linear and approximately could be represented by these computations:

Si (CFU) _550 nm_ = OD × 7.5 × 10^9^ (1)

Ss (CFU) _550 nm_ = OD × 5.5 × 10^9^ (2)

With an optical density from 0.3 to 0.8 (550 nm).

### 4.5. RNA/DNA/Protein Extraction from Bactecria Deposited on Cylinder Surface

RNA, DNA and protein extractions were simultaneously carried out for each cylinder, using a modified TRIzol reagent method (TRIzol^®^, Life Technologies, Carlsbad, CA, USA). Each cylinder was immediately immersed in an Eppendorf tube containing 0.01 mL of TRIzol, following the procedure described by Xiong et al. [68]. The extracts (RNA, DNA, proteins) were stored at −80°C prior to analysis.

### 4.6. Protein Quantification on the Cylinder Surface

The total protein concentration on the implant surface was obtained at different times from the protein extracts with the Warburg’s method [69] and then measured at A_260_ nm and A_280_ where A_280_ nm value had to be greater than the A_260_ nm. Total protein concentration was expressed as mg/cm^2^ of the titanium cylinder surface.

A serial 10-fold serial dilution of albumin bovine (from 100 to 1 mg/mL) (Merck Sigma-Aldrich, Darmstadt, Germany) was performed to calculate the standard error and the sensitivity of the procedure (Figure 2).

### 4.7. Bacterial Count by Real-Time PCR

The total mass of *S. intermedius* and *S. salivarius* on the cylinder surface was evaluated through the modified method already described for *S. faecalis* biofilm by Denotti et al. [62] by using a Real-Time PCR (RT-PCR) method. The oligonucleotide primers used in this work were designed following the bioinformatic procedure described by Arcadu et al. [70] (Table 2). The total biofilm amount on the cylinder surface was measured by using 0.02 mL of extracted DNA. Total bacterial count was performed using two different primers sets (OG439-OG440 and OG437-OG438) to quantify *S. salivarius* and *S. intermedius*, respectively. RT-PCR reaction was conducted with a Light Cycler (Roche Diagnostics, Mannheim, Germany) and SYBR Premix Ex Taq Kit (Takara Bio, Mountain View, CA, USA). For each analysis, three biological replicates were carried out, and data were expressed as mean ± SD. Threshold Cycle (CT) units comprising ± 0.9 of the mean were considered significant. The bacterial titre was expressed as [bacterial genomes/µL] by the interpolation of the sample’s threshold cycle with a standard curve obtained with previously 10-fold diluted *S. intermedius* DNA, from 10^2^ to 10^7^ genomes/µL (see supplementary material Supplementary File S1 and Supplementary File S2).

### 4.8. luxS Expression Pattern as Biofilm Formation Marker

Bacterial RNA was reverse-transcribed to cDNA using ImProm-II Reverse Transcriptase Kit (Promega, Madison, WI, USA) and quantitative RT-PCR was performed with Light Cycler DNA-Master SYBR Green I Kit (Roche, Mannheim, Germany). The relative gene expression was analysed using the 2-ΔΔCT method [71]; 16S rRNA *S. intermedius* was chosen as reference gene while the expression of *luxS* gene was quantified as biofilm maker formation with species-specific primers: OG 437 and OG438 listed in Table 1. In addition, the use of a relative quantitation to measure the *luxS* mRNA amount avoid the possible experimental artifacts due to differences in bacterial cell numbers [62].

### 4.9. Statistical Analysis

Quantitative RT-PCR data were expressed as mean ± SD. Values of fold change in gene expression above 2 or below 0.5 were considered significant. The difference in adhesion capacity to the cylinder of the DIB between the two strains was evaluated with Student’s t-test. To compare the groups of bacterial species within the same group of the cylinder surface and the differences between specimen surfaces within the same species a Two-way analysis of variance (ANOVA) was used (α = 0.05) (GraphPad InStat software, San Diego, CA, USA) [72].

## 5. Conclusions

This study describes a preliminary approach to evaluate the interaction of *S. salivarius* K12 in a monomicrobial in vitro model of peri-implantitis. These first results suggest a fast development of the biological events during the biofilm formation on a titanium cylinder surface. Under the conditions used in this work, the “biofilm structuration window” is related to *S. intermedius* growth in the first 6–12 h, in accordance with other experiments described in the literature on *Streptococcus* spp. These results could be interesting in clinical practice in in vivo peri-implantitis treatment for two reasons:

(i) The time of action to avoid biofilm formation could be extremely short and an effective appropriate prophylactic action could be necessary;

(ii) The use of bacteriotherapy with *S. salivarius* could be a new approach in the treatment of biofilm-related implant diseases, as well as periodontitis.

However, further investigations are needed to explain the exact interaction mechanisms, for example, by using periimplantitis polymicrobial biofilm and additional measurements as AI-2 production in probiotic and in biofilm, and a precise study is required on the role of salivary proteins in biofilm–probiotic behaviour. In addition, our research describes a first approach to conduct further comparative studies with commercial products, i.e., titanium implants with different designs and surface’s roughness.

## Figures and Tables

**Table 1:** Antimicrobial activity of *S. salivarius* filtrate medium against *S. intermedius*.

Filtrate	MIC	MBC	MBIC
	Medium (%)		
*S. salivarius* medium	50	>50	12.5
Control	>50	>50	>50

**Table 2:** Oligonucleotides used in this work for bacteria count and for gene expression assay.

Oligo Name	Oligo Sequence 5′–3′	Oligo Name	Gene Name GenBank Accession	bp
*S. salivarius*	GTAAAGCTCTGTTGTAAGTC	OG439	*16S rRNA* AY692453	600
	AACTTTCTATCTCTAGAAATA	OG440		
*S. intermedius*	GTAAAGCTCTGTTGTTAAGG	OG437	*16S rRNA* AF104671	600
	AAAGCTCTATCTCTAGAGCGG	OG438		
*S. intermedius*	ATTGTCAAAGCCCCTTAT	OG349	*luxS* DQ836241	266

## Supplementary Materials

The following are available online at https://www.mdpi.com/2076-0817/9/12/1069/s1, File S1: *S. intermedius* LuxS expression pattern design, File S2: PCR Oligos design for absolute quantification of *S. salivarius* and *S. intermedius*.

## Data Accessibility

All data are available in the main text or electronic supplementary material. Link/footnotes Center for Biofilm Engineering: http://www.biofilm.montana.edu/.
