# Supplementary material for: In vitro Interactions between Streptococcus intermedius and Streptococcus salivarius K12 on a Titanium Cylindrical Surface
Source: Pathogens. 2020 Dec 20;9(12):1069. doi: 10.3390/pathogens9121069 (PMC7765831; doi:10.3390/pathogens9121069)
Supplement: Supplementary file 1 [file pathogens-09-01069-s001.zip › File S1_ LuxS expression pattern.pdf]

S. intermedius LuxS expression pattern design

Streptococcus intermedius strain NCTC 11324
S-ribosylhomocysteine lyase (luxS) gene, GeneBank accession n. DQ836241

```
ATAATTTATTAAAAACAAAGGAGTTTTCTGATGGTAAAAGAAGTAATAGTTGAAAGTTTTGAGTTAGACC
ACACGATTGTCAAAGCCCCTTATGTGCGTTTGATTGGCGAAGAAATTGGTCCAAAAGGTGATGTCATCTC
GAATTTTGATATTCGTCTTGTCCAACCTAACGAGGATTCTATCCCAACTGCTGGACTACACACAATTGAA
CATCTCCTAGCAAAGCTCATTCGCACTCGGATTGATGGCATGATTGATTGCTCCCCTTTTGGCTGCCGTA
CTGGCTTTCACATGATCATGTGGGGACGACATAGTACAACCGAAATTGCCAAAGTCATAAAGGCCTCTTT
AGAGGAAATAGCTGGAGAAACCACCTGGACTGATGTCCCTGGCACAACCATTGAATCCTGTGGAAATTAT
AAAGACCACAGTCTCTTTTCTGCCAAAGAATGGTGCAAGCTCATTTTAGATCAAGGAATCTCTGACGATC
CTTTTAAACGCCATCTTGTATAA
```

PCR Oligos Thermodynamic parameters

Oligo name	Sequence 5’-----------------------3’	Length bp/Tm °C*	PCR parameters
**OG-349**	ATTGTCAAAGCCCCTTAT	18/56.0 °C	Ta= 50,7°C<br>44,0 GC%
**OG-350**	TTTATGACTTTGGCAATTTC	20/57.5 °C	Amplicon length = 266 bp

- ✓ *Calculated according to Nearest Neighbor method
- ✓ Salt Concentration = [50 mM]
- ✓ PCR efficiency = 98%

Real time PCR conditions

The real time PCR relative quantitation was performed with a LightCycler instrument (Roche Diagnostics Mannheim, Germany) and a SYBR Premix Ex Taq Kit (TaKara-Clontech®) according to the manufacturer's instructions. The 0.02 mL final volume contained: 0.0064 mL of Dnase-RNase free water, 0.01 mL Premix Ex Taq, 0.0008 mL of a SYBR Green 1/10000 solution 1 μM of each primer, 0.0004 mL, and 0.002 mL of cDNA extract. The PCR program was: (i) denaturation at 95°C for 30 secs, (ii) 40 cycles of: 5 secs at 95°C, 30 secs at 60°C, 20 secs at 80°C. The melting curve was performed for 0 seconds at 95°C, 45°C, 95°C. Transition rates were: 10°C/s in 72°C segment, 0.1°C/s in 45°C segment and 20°C/s for other steps. Fluorescence was detected at the end of the 80 °C segment in the PCR step (single mode), and at 45°C segment in the melting step (continuous mode) in the F1 channel. During the initial optimization of the real-time PCR reaction, products were analysed by using agarose gel and real time PCR melting curve analysis to ensure a correct sample product size and the melting temperature (Tm) of each amplicon. PCR efficiency was evaluated by using each standard curve slope with this equation:

$[e = 10^{-1/slope}]$

Where: e = theoretical efficiency, Slope = the slope of the standard curve, plotted with the y axis as Threshold cycle (Ct) and the x axis as log known quantity of *S. intermedius* DNA (from $1*10^{-3}$ to 10 ng).

References

Kubista, M., Andrade, J. M., Bengtsson, M., Forootan, A., Jonak, J., Lind, K., Sindelka, R., Sjoback, R., Sjogreen, B., Strombom, L., *et al.* 2006 The real-time polymerase chain reaction. *Mol Aspects Med*. **27**, 95-125. (10.1016/j.mam.2005.12.007)

Footnotes/Web/Bioinformatic tools/

- **Reverse complement calculator**

http://reverse-complement.com/

- **Oligo7**

https://www.oligo.net/downloads.html
