# Supplementary material for: In vitro Interactions between Streptococcus intermedius and Streptococcus salivarius K12 on a Titanium Cylindrical Surface
Source: Pathogens. 2020 Dec 20;9(12):1069. doi: 10.3390/pathogens9121069 (PMC7765831; doi:10.3390/pathogens9121069)
Supplement: Supplementary file 1 [file pathogens-09-01069-s001.zip › File S2_PCR Oligos design for absolute quantification.pdf]

PCR Oligos design for absolute quantification of *S. salivarius and S. intermedius*

The PCR target region was identified by multiple sequence alignment programs (Clustal omega) by means of the complete 16S rRNA gene sequences, available in the DNA data Bank (GenBank). The selected sequence regions (Figure 1), showed in 3' region a differential nucleotide motif between *S, intermedius and S. salivarius* K12, these were able to discriminate the two microbial species. In these conditions the PCR real time reaction amplified a 600 base pair amplicon and an annealing temperature of 50°C for both strains (Table I). The theoretical melting temperatures of the primers (Tm_s), the formation of oligonucleotide dimers and self-complementarity were evaluated using Oligo Program version 7 (MedProbe, Oslo, Norway) and by DINA melt Web server for DNA Hybridisation prediction, homodimer simulation with salt conditon to 2 mM [Mg^{2+}] and 50 mM [Na^+]. Real time PCR reactions were performed by using a Light Cycler instruments ver. 2.2 and SYBR Premix Ex Taq Kit (TaKara-Clontech®) according to manufacturer's instructions. The real time PCR standard curve was made on DNA extracts, obtained from different *S. salivarius and S. intermedius* suspensions with a concentration range from 10^7 to 10^2 DNA genomes/2μl. We have calculated that the two bacterial genomes, approximately, were inside these parameters : (i) genome length = 2.19 Mb for *S. salivarius* and 1.94 Mb for *S. intermedius* (NCBI-genome, median values), (ii) we have used for mass conversion this subsequent computational formula, Dolzel *et al.*, 2003,

$$[\ bp = M *0.978*10^6] \neg [M = bp*0.978*10\text{-}^6]$$ were:

M = DNA mass [fg]

bp = base pair of DNA fragment

Thus 1.7 in and 1.5 femtograms/genome respectively for *S. salivarius* and *S. intermedius*

The amount of bacterial genomes was calculated by an interpolated threshold cycle with a standard curve performed for each strain, as example figure 3 *S. salivarius* amplification curve, figure 4 standard curves for both bacterium.

Fig. 1. 16S rRNA, nucleotide sequence and position of the PCR oligos.

```
S.intermedius      TTTTCGGATCGTAAAGCTCTGTTGTTAAGGAAGAACGAGTGTGAGAATGGAAAGTTCATA      480
S.salivarius       TTTTCGGATCGTAAAGCTCTGTTGTAAGTCAAGAACGAGTGTGAGAGTGGAAAGTTCACA      428
                   ************************* *   **************** *********** *

S.intermedius      CTGTGACGGTACTTAACCAGAAAGGGACGGCTAACTACGTGCCAGCAGCCGCGGTAATAC      540
S.salivarius       CTGTGACGGTAGCTTACCAGAAAGGGACGGCTAACTACGTGCCAGCAGCCGCGGTAATAC      488
                   ***********  * *********************************************

S.intermedius      GTAGGTCCCGAGCGTTGTCCGGATTTATTGGGCGTAAAGCGAGCGCAGGCGGTTAGATAA      600
S.salivarius       GTAGGTCCCGAGCGTTGTCCGGATTTATTGGGCGTAAAGCGAGCGCAGGCGGTTTGATAA      548
                   ****************************************************** *****

S.intermedius      GTCTGAAGTTAAAGGCAGTGGCTCAACCATTGTAGGCTTTGGAAACTGTTTAACTTGAGT      660
S.salivarius       GTCTGAAGTTAAAGGCTGTGGCTCAACCATAGTTCGCTTTGGAAACTGTCAAACTTGAGT      608
                   **************** ************* **  **************  *********

S.intermedius      GCAGAAGGGGAGAGTGGAATTCCATGTGTAGCGGTGAAATGCGTAGATATATGGAGGAAC      720
S.salivarius       GCAGAAGGGGAGAGTGGAATTCCATGTGTAGCGGTGAAATGCGTAGATATATGGAGGAAC      668
                   ************************************************************

S.intermedius      ACCGGTGGCGAAAGCGGCTCTCTGGTCTGTAACTGACGCTGAGGCTCGAAAGCGTGGGGA      780
S.salivarius       ACCGGTGGCGAAAGCGGCTCTCTGGTCTGTAACTGACGCTGAGGCTCGAAAGCGTGGGGA      728
                   ************************************************************

S.intermedius      GCGAACAGGATTAGATACCCTGGTAGTCCACGCCGTAAACGATGAGTGCTAGGTGTTAGG      840
S.salivarius       GCGAACAGGATTAGATACCCTGGTAGTCCACGCCGTAAACGATGAGTGCTAGGTGTTGGA      788
                    ********************************************************* *

S.intermedius      TCCTTTCCGGGACTTAGTGCCGCAGCTAACGCATTAAGCACTCCGCCTGGGGAGTACGAC      900
S.salivarius       TCCTTTCCGGGATTCAGTGCCGCAGCTAACGCATTAAGCACTCCGCCTGGGGAGTACGAC      848
                   ************ * *********************************************

S.intermedius      CGCAAGGTTGAAACTCAAAGGAATTGACGGGGGCCCGCACAAGCGGTGGAGCATGTGGTT      960
S.salivarius       CGCAAGGTTGAAACTCAAAGGAATTGACGGGGGCCCGCACAAGCGGTGGAGCATGTGGTT      908
                   ************************************************************

S.intermedius      TAATTCGAAGCAACGCGAAGAACCTTACCAGGTCTTGACATCCCGATGCCCGCTCTAGAG      1020
S.salivarius       TAATTCGAAGCAACGCGAAGAACCTTACCAGGTCTTGACATCCCGATGCTATTTCTAGAG      968
                   *************************************************    *******

S.intermedius      ATAGAGCTTTACTTCGGTACATCGGTGACAGGTGGTGCATGGTTGTCGTCAGCTCGTGTC      1080
S.salivarius       ATAGAAAGTTACTTCGGTACATCGGTGACAGGTGGTGCATGGTTGTCGTCAGCTCGTGTC      1028
                   *****   ****************************************************
```

Accession numbers:

GenBank: FJ154804.1

GenBank: AF104671.1

Tab. I. Nucleotide sequence of the PCR oligos used in this work for absolute quantitation.

Oligo name	Oligo sequence 5'---3'	Oligo name	Gene name GenBank accession	bp
S. salivarius	GTAAAGCTCTGTTGTAAGTC<br>AACTTTCTATCTCTAGAAATA	OG439<br>OG440	16S rRNA<br>FJ154804	600
S. intermedius	GTAAAGCTCTGTTGTTAAGG<br>AAAGCTCTATCTCTAGAGCGG	OG437<br>OG438	16S rRNA<br>AF104671	600

Annealing temperature = 50° C

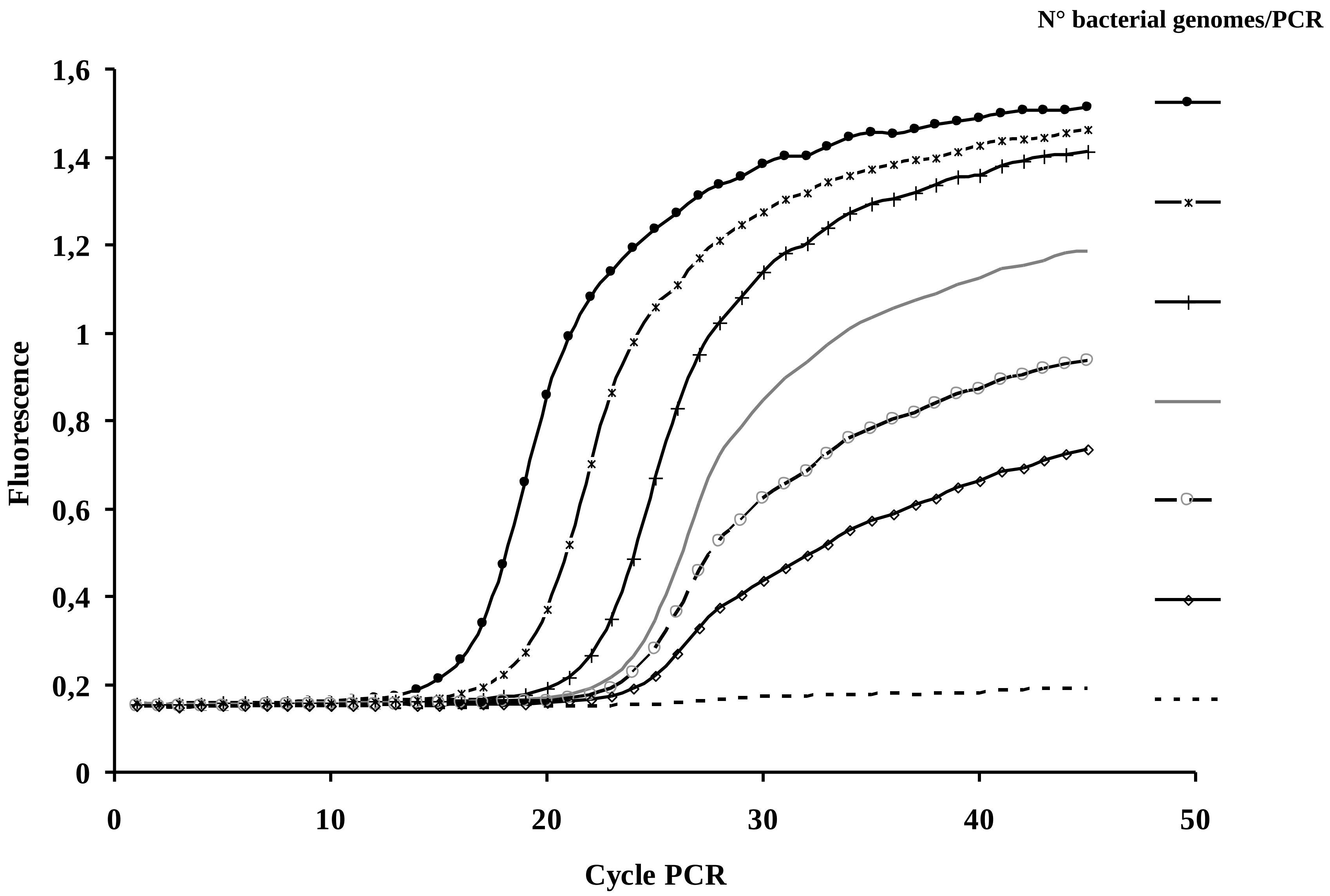

Fig. 3. *S. salivarius* K12 amplification curve, obtained with bacterial suspensions ranged from 10^2 to 10^7 genomes /2 µl for each strain.

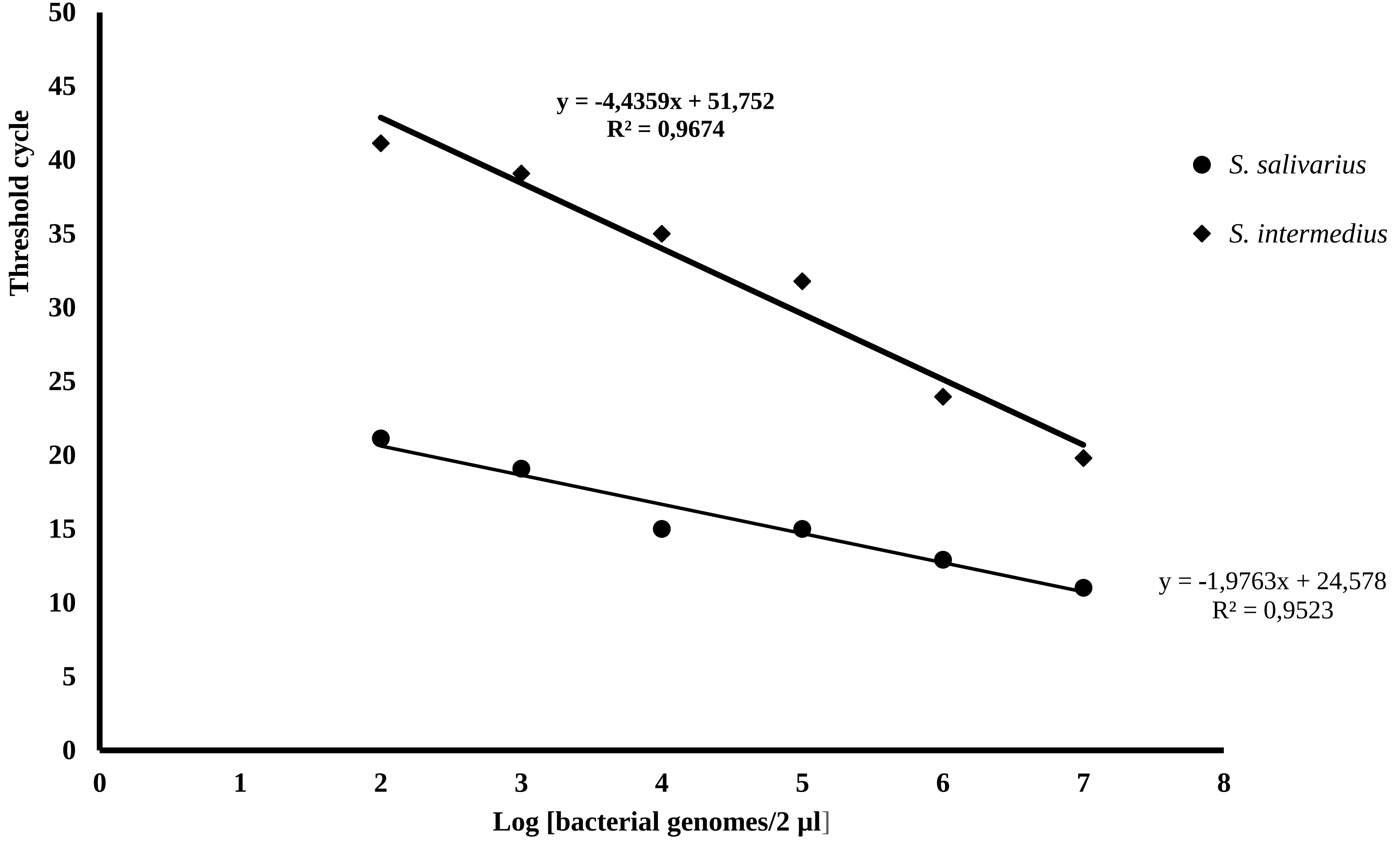

Fig.4. *S. intermedius* and *S. salivarius* standard curves obtained with bacterial suspensions ranged from 10^2 to 10^7 genomes /2 µl for each strain.

References

Dolezel J, Bartos J, Voglmayr H, Greilhuber J. (2003). Nuclear DNA content and genome size of trout and human. Cytometry, 51:127-8.

Markham, N. R. & Zuker, M. (2008) UNAFold: software for nucleic acid folding and hybridization. In Keith, J. M., editor, Bioinformatics, Volume II. Structure, Function and Applications, number 453 in Methods in Molecular Biology, chapter 1, pages 3–31. Humana Press, Totowa, NJ.

Arcadu B, Orrù M, Piga R, Orrù G. (2012). Designing of sequencing assay assisted by capillary electrophoresis based on DNA folding analysis: an application to the VCAM1 gene.
Electrophoresis, 33:1215-9. doi: 10.1002/elps.201100590.

Footnotes

DNA calculator MW/length : http://www.calctool.org/CALC/prof/bio/dna

Clustal omega : https://www.ebi.ac.uk/Tools/msa/clustalo/

Genome-NCBI: (*S. intermedius*): https://www.ncbi.nlm.nih.gov/genome/?term=streptococcus++intermedius
(*S. salivarius*): https://www.ncbi.nlm.nih.gov/genome/?term=streptococcus+salivarius

The DINAMelt Web Server: http://unafold.rna.albany.edu/?q=DINAMelt/Homodimer-simulations

Reverse complement calculator : http://reverse-complement.com/

Oligo7: https://www.oligo.net/downloads.html
